# Enhanced insulin receptor interaction by a bifunctional insulin-transferrin fusion protein: an approach to overcome insulin resistance

**DOI:** 10.1038/s41598-020-64731-9

**Published:** 2020-05-07

**Authors:** Yuqian Liu, Hsuan-Yao Wang, Juntang Shao, Jennica L. Zaro, Wei-Chiang Shen

**Affiliations:** 1 Department of Pharmacology and Pharmaceutical Sciences, University of Southern California School of Pharmacy, 1985 Zonal Ave, Los Angeles, CA 90089-9121 USA; 2 School of Basic Medical Sciences, Anhui Medical University, Hefei, 230032 China

**Keywords:** Pharmaceutics, Molecular medicine

## Abstract

Bifunctional fusion protein design has been widely utilized as a strategy to increase the efficacy of protein therapeutics. Previously, we proposed a novel application of the bifunctional fusion protein design through the introduction of proinsulin-transferrin (ProINS-Tf) fusion protein as a liver-specific protein prodrug to achieve a glucose-lowering effect in type 1 diabetic mice. In this report, we studied the binding characteristics of this activated fusion protein to the insulin receptor to elucidate its mechanism in eliciting insulin receptor-mediated signaling. We found that, with the assistance of the transferrin moiety binding to the transferrin receptor, the activated ProINS-Tf exhibited significantly higher binding affinity to the insulin receptor compared with the native insulin, resulting in a prolonged and stronger Akt phosphorylation. This enhanced induction by activated ProINS-Tf overcame insulin resistance in palmitate-treated HepG2 cells. ProINS-Tf also demonstrated a better glucose-lowering effect than native insulin, even with a much lower dose and less frequent injections, in non-obese diabetic mice with insulin resistance symptoms. The activated ProINS-Tf, serving as a bivalent protein molecule, could be a new insulin analog to overcome insulin resistance, which is associated with several diseases, including type 2 diabetes and non-alcoholic fatty liver disease.

## Introduction

Insulin (INS) resistance is the major cause of the development of type 2 diabetes (T2D), and it is often referred as a state in which a higher than normal level of INS is required to achieve the normal response^1–3^. INS resistance may result from alterations at different cellular levels, including insulin deficient signaling, inflammation, endoplasmic reticulum stress, and mitochondrial dysfunction^3^. Type 2 diabetic patients often require intensive insulin treatment to maintain glycemic control, which leads to increased risk of hypoglycemia, weight gain, and further deterioration of INS resistance state^1,4^. Previously, INS X10 with higher IR binding affinity have been studied as a rapid-acting INS analogue to treat type 2 diabetes^5^. INS X10 demonstrated sustained effect in inducing IR-mediated signaling; however, the development of INS X10 was discontinued due to disproportionate increase in mitogenic activity and higher breast cancer incidence in the long-term rat studies^6^. It is still a great challenge to develop novel INS analogues with enhanced binding affinity to the IR to overcome INS resistance safely and effectively.

A proinsulin-transferrin (ProINS-Tf) fusion protein was previously developed as a novel long-acting and liver-targeted INS prodrug for treating type 1 diabetic (T1D) mice^7,8^. Proinsulin (ProINS), as a precursor of insulin (INS), has a much lower binding affinity to INS receptor (IR) and the resultant biologic potency is only 1% or less relative to INS^9^. Therefore, ProINS-Tf is initially inactive and requires a lag time to be activated before exhibiting activity in stimulating Akt phosphorylation in H4IIE cells^10^. The long-acting glucose-lowering effect of ProINS-Tf observed *in vivo* therefore could be attributed to the activated form of the fusion protein, i.e. immune-reactive INS-Tf (irINS-Tf), which is detectable with INS-specific radioimmunoassay^10^. This activated fusion protein can be considered as a bifunctional fusion protein, containing the two functional moieties of INS and Tf. By combining two protein domains together, bifunctional fusion proteins exhibit the properties of each of the functional moiety and therefore can achieve an improved therapeutic profile^11–13^. Pharmacokinetics (PK) and pharmacodynamics (PD) studies of bifunctional Tf fusion proteins suggested that the addition of the Tf domain to protein drugs, including human growth hormone and granulocyte colony stimulating factor, preserved the physiological functions of the protein drug domain while increasing its plasma half-life via the Tf domain^14^. As a bifunctional protein with two active binding groups, irINS-Tf should be active on both INS receptor (IR) and Tf receptor (TfR).

In this report, we demonstrate that irINS-Tf exhibits an increased affinity and retention of INS binding to IR. The enhanced binding effect is most likely attributed to the bivalent binding of this INS analog to both IR and TfR in liver cells. Due to the high affinity and retention to IR, irINS-Tf is capable of overcoming INS resistance in insulin-resistant HepG2 cells and in severe hyperglycemic NOD mice.

## Materials and Methods

### Materials

Recombinant human INS and apo-Tf were purchased from Sigma (St. Louis, MO). INS was dissolved in 0.1 M HCl and further diluted in PBS to desired concentrations. Apo-Tf was saturated with iron by incubating with ferric ammonium citrate followed by dialysis in PBS. Receptor grade radioactive INS labeled with ^125^I on tyrosineA^14^ was purchased from Perkin Elmer (Waltham, MA).

### Cell cultures

Human embryonic kidney HEK293, rat hepatoma H4IIE, human hepatocellular carcinoma HepG2, and human mammary gland adenocarcinoma MCF-7 cell lines were purchased from ATCC (Manassas, VA). HEK293, H4IIE, and HepG2 cells were cultured in Dulbecco’s modified Eagle’s medium (DMEM) supplemented with 10% fetal bovine serum (FBS) and 0.1 unit of penicillin/0.1 mg streptomycin (Invitrogen) per mL. MCF7 cells were cultured in Roswell Park Memorial Institute (RPMI) 1640 medium supplemented with 10% FBS, 0.01 mg human INS, and 0.1 unit of penicillin/0.1 mg streptomycin per mL All cells were maintained in a 5% CO_2_ humidified incubator at 37 °C. Confluent cells were subcultured using 0.05% trypsin-EDTA on a regular basis.

### Protein preparation

ProINS-Tf fusion protein was produced and purified as previously described^10^. Briefly, DNA plasmids containing the ProINS-Tf fusion gene with His-tag at c-terminus were transfected into HEK293 cells (ATCC, Manassas, VA). Fusion protein was collected from conditioned medium, concentrated using tangential flow filtration systems (Millipore, Billerica, MA), and further purified by applying through Ni-NTA chromatography. Tf and ProINS moieties were both confirmed in the purified fusion protein by recognition of anti-Tf and anti-ProINS antibodies, respectively, in Western blot assay.

### Active form of ProINS-Tf: irINS-Tf

The active form of the fusion protein, irINS-Tf, was generated by subjecting ProINS-Tf to H4IIE cell-mediated conversion prior to the cell-based assays^10^. In brief, ProINS-Tf (10 nM) was activated by incubating with confluent H4IIE cell monolayers for 24 h. The irINS-Tf doses shown in this report represent the concentration of the active form as determined by using a highly INS specific RIA with less than 0.2% cross-reactivity to ProINS^10^.

### Akt phosphorylation assays

The cell-based mechanistic studies of the fusion protein were carried out in HepG2 cells, which express extremely low levels of hepatic metabolism enzymes and altered TfR recycling mechanism^15,16^, and are ineffective at converting ProINS-Tf to its active form^17^. Therefore, the prodrug form of the fusion protein, ProINS-Tf, is inactive in Akt signaling in this cell line.

Confluent HepG2 cells were starved in serum-free medium for 16–18 h before the experiment, and starved cells were then treated with different proteins for the indicated period of time. After treatment, cells were washed and lysed with cell lysis buffer (Cell Signaling Technology) supplemented with protease and phosphatases inhibitor cocktail (Thermo Scientific) on ice. After BCA quantification, equal amounts of total cellular proteins from each treatment group were subjected to Western blot analysis using anti-phospho-Akt antibody (Ser473, Cell Signaling) and anti-GAPDH antibody (D16H11, Cell Signaling). Band densities were quantified by Image Lab software (Bio-Rad, Hercules, CA).

For pulse-chase Akt phosphorylation assays, starved HepG2 cells were first pulsed with dosing medium containing different proteins for 30 min at 4 °C. Cells were then washed 3 times with ice-cold PBS and chased by incubation in protein-free DMEM medium at 37 °C for the indicated period of time.

### MCF-7 cells proliferation assay

MCF-7 Cells were seeded in triplicates in 96-well plates (5,000 cells per well). Cells after 24 h serum starvation were treated with 10 nM of human INS, holo-Tf or ProINS-Tf in phenol red free RPMI medium supplemented with 0.1% BSA (day 1). This treatment was repeated in two consecutive days (day 2 and day 3). On day 4, cells were incubated for 4 h at 37 °C with 0.5 mg/ml 3-(4,5-dimeth- ylthiazol-2-yl)-2,5-diphenyltetrazolium bromide (MTT) prior to cell lysis by acidified isopropanol (4 mM HCl, 0.1% Nondet P-40). Formazan dye production was measured using Infinite® F200 (TECAN) at 570 nM. Absorbance readings were normalized with control group cultured in 0.1% BSA RPMI and the OD value was referred as 100%. For the H4IIE cell pretreated set of protein treatment, 10 nM of the aforementioned protein was incubated with confluent H4IIE cells for 24 h. Subsequently, the supernatant of the conditioned medium was collected and applied to MCF-7 cells for proliferation assays.

### Insulin resistance in HepG2 cells induced by palmitate treatment

Bovine serum albumin (BSA)-palmitate complex preparation was adapted from previous studies^18,19^. In brief, 8% BSA and 8 mM sodium palmitate were dissolved separately in 150 mM NaCl solution. The two solutions were mixed at a 1:1 ratio and stirred for 1 h at 37 °C to produce a stock complex solution of 4% BSA/4 mM palmitate. To induce INS resistance in HepG2 cells, cells were treated in BSA/palmitate complex diluted in serum-free DMEM for 16–18 h and then utilized for Akt phosphorylation assays.

### Insulin receptor binding assays

To prepare the activated fusion protein for the binding assay, ProINS-Tf (10 nM) was first incubated with confluent H4IIE cells for 24 h, and the conditioned medium containing partially activated ProINS-Tf was serially diluted to subsequent lower concentrations, or concentrated by 10-fold to get a higher concentration of the active form. For dosing solutions preparation, ^125^I-Tyr(A14)-INS was mixed with serial concentrations of INS, ProINS-Tf, or irINS-Tf in DMEM containing 0.1% BSA with or without 100-fold free Tf. HepG2 cells were seeded in 24-well plates and allowed to reach confluence before each experiment. Prior to dosing, cells were incubated in DMEM with 0.1% BSA at 37 °C for 30 mins to reach the equilibration and depletion of residual serum components. Cells were then washed three times with PBS and dosed with ice-cold dosing solutions containing ^125^I-Tyr(A14)-INS and various concentrations of proteins for 2 h at 4 °C. After incubation, cells were washed three times with ice-cold PBS and then dissolved in 1 N NaOH. Radioactivity associated with dissolved cell monolayers was counted by the Packard Cobra II gamma counter and normalized to the amount of total cellular proteins. Binding curves and corresponding IC_50_ values were generated using GraphPad Prism software by fitting the data with the non-linear regression model of one-site competitive binding.

### Animals

Female NOD/ShiLtJ mice (8 weeks old) were purchased from Jackson Laboratory (Bar Harbor, ME). Mice were housed at 12 h light/12 h dark cycles at room temperature 22 ± 3 °C and relative humidity 50 ± 20%. All mice had access to a regular rodent diet (Labdiet, St. Louis, MO) and water ad libitum. Blood glucose (BG) levels of NOD mice were measured through tail vein blood sampling weekly using a OneTouch glucose meter (LifeScan, Milpitas, CA) with detection range between 20 and 600 mg/dL to monitor the development of hyperglycemia. NOD mice were classified as moderately or severely hyperglycemic based on two consecutive non-fasting BG measurements between 200 and 300 mg/dL or greater than 550 mg/dL, respectively. All animal studies were conducted in accordance with NIH guidance and approved by the University of Southern California Institutional Animal Care and Use Committee.

### Meal challenge study

NOD mice with severe hyperglycemia were randomly divided into three groups (n = 4 for each group), and the mice were subcutaneously injected with either a single injection of ProINS-Tf (dose: 45 nmol/kg) before the start of the experiment, or two injections of INS (dose: 180 nmol/kg) or PBS before the start of each feeding period. The blood glucose level was monitored in two 2 h feeding/6 h fasting cycles.

### Statistical analysis

Data are represented as mean ± standard deviation (n ≥ 3) for all experiments. Two-tailed Student’s t-test was used to compare between 2 groups of results, and one-way ANOVA was used to compare among 3 or more groups. Differences were regarded as statistically significant when p < 0.05.

## Results

### Active irINS-Tf demonstrated enhanced and sustained effect on activating Akt signaling

**Figure 1:**
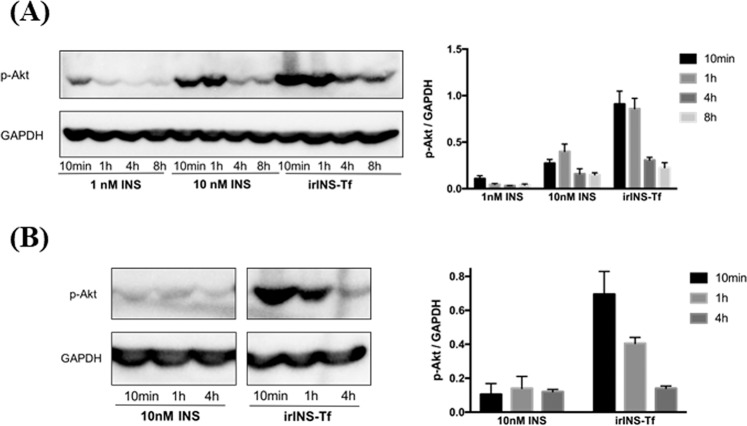
Active irINS-Tf exhibited enhanced and prolonged effect on inducing Akt phosphorylation in HepG2 cells. (**A**) Time-course Akt phosphorylation in HepG2 cells. Starved HepG2 cells were treated for indicated period of time with 1 nM of INS, 10 nM of INS, or irINS-Tf converted from10 nM of ProINS-Tf. (**B**) Pulse-chase Akt phosphorylation assay in HepG2 cells. Starved HepG2 cells were treated with ice-cold dosing solution containing 10 nM of INS or irINS-Tf converted from 10 nM of ProINS-Tf for 30 min at 4 °C, and then chased in DMEM only medium for indicated period of time at 37 °C. Phospho-Akt band density was normalized with corresponding GAPDH band density. Data represents average values with error bars indicating the standard deviation (N = 3).

Compared to native INS, irINS-Tf demonstrated an increased Akt phosphorylation at the onset of treatment (10 min), suggesting its enhanced ability on activating IR regulated pathway (Fig. 1A). In the prolonged treatment times, 1 h, 4 h or 8 h, the active fusion protein also caused a higher level of Akt phosphorylation compared to the INS group (Fig. 1A), indicating a sustained IR binding effect.

During the pulse-chase assays (Fig. 1B), the cell associated irINS-Tf caused significantly more Akt phosphorylation at 10 min and 1 h during the chase period, while INS demonstrated very low activity. After 4 h of chasing, the p-Akt level stimulated by irINS-Tf decreased to a similar level as of INS.

### INS receptor binding of INS, ProINS-Tf, and irINS-Tf

**Figure 2:**
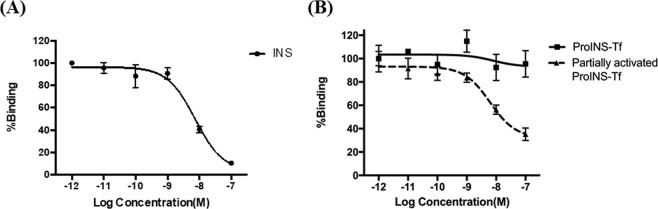
IR binding profiles of (**A**) INS and (**B**) ProINS-Tf (before and after activation) on HepG2 cells. INS was directly applied to the binding assay. ProINS-Tf was either directly applied to the binding assay, or pre-incubated with H4IIE cells for activation and then applied to the binding assay. Following binding at 4 °C for 2 h, the cell-associated radioactivity was counted and normalized by total cell protein. Data were presented as an average of percentage of binding relative to the binding of the lowest concentration (N = 3). IC_50_ values were estimated for INS and ProINS-Tf using GraphPad Prism. IC_50_ values: INS, 7.29 nM; ProINS-Tf,>> 100 nM; activated ProINS-Tf, 18.54 nM.

Relative IR binding affinities were measured for both INS and ProINS-Tf on HepG2 cell monolayers. Compared to INS (IC_50_ = 7.29 nm, Fig. 2A), ProINS-Tf demonstrated a very weak binding affinity to IR (IC_50_ >> 100 nM, Fig. 2B). The partially activated irINS-Tf showed significantly increased binding affinity to IR, with an IC_50_ of 18.54 nM (Fig. 2B), suggesting the conversion of the ProINS moiety to a much more active INS form that had higher binding affinity to IR. However, in this experiment, only 6.7% of the H4IIE cell-activated ProINS-Tf was converted to irINS-Tf as determined by INS specific RIA. Based on the amount of irINS-Tf present in the dosing media, the IC_50_ of pure irINS-Tf can be estimated as 1.24 nM.

### Excess Tf interfered with the binding of activated ProINS-Tf

**Figure 3:**
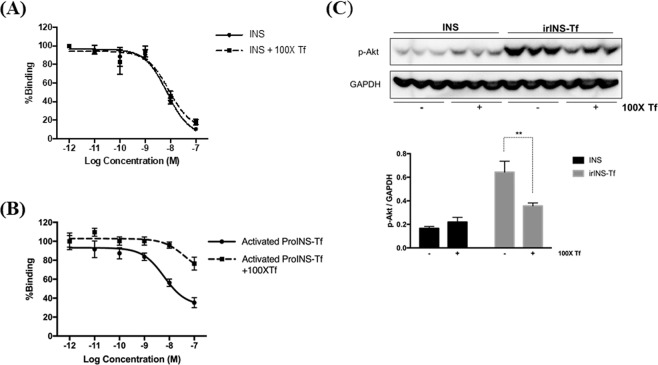
Effect of Tf competition on the IR binding of INS and irINS-Tf in HepG2 cells. (**A**) IR binding profile of INS. (**B**) IR binding profile of activated ProINS-Tf. At each concentration, 100-fold of Tf was added to compete for TfR binding. ProINS-Tf was pre-incubated with H4IIE cells for activation and then applied to the binding assay. Following binding at 4 °C for 2 h, the cell-associated radioactivity was counted and normalized by total cell protein. Data were presented as an average of percentage of binding relative to the binding of the lowest protein concentration (N = 3). IC_50_ values estimated for INS and activated ProINS-Tf with 100-fold Tf competition were 9.87 nM and 321.5 nM, respectively. (**C**) Akt phosphorylation assay with Tf competition in HepG2 cells. Starved HepG2 cells were treated for 10 min with 10 nM of INS or irINS-Tf converted from 10 nM of ProINS-Tf, with or without addition of 100-fold Tf (1μM). Phospho-Akt band density was normalized with corresponding GAPDH band density. Data were presented as the average values with error bars indicating the standard deviation (N = 3). **Indicated *p* < 0.01.

The IR binding assay was carried out for INS and irINS-Tf in the presence of 100-fold excess Tf at each protein concentration. The binding profile of INS was not majorly affected by addition of excess Tf, with IC_50_ changed from 7.29 nM to 9.87 nM (Fig. 3A). The IR binding affinity of H4IIE cell-activated ProINS-Tf was significantly decreased in the presence of 100-fold excess Tf (Fig. 3B). The IC_50_ of activated ProINS-Tf increased from 18.54 nM to 321.5 nM, corresponding to an approximate 17-fold decrease in IR binding affinity.

The effect of Tf on the IR binding affinity of irINS-Tf was further investigated by including a high concentration of Tf in a short-term Akt phosphorylation assay. As shown in Fig. 3C, with addition of 100-fold excess Tf (1 μM), Akt phosphorylation level induced by 10 nM INS was not significantly altered. For irINS-Tf, on the other hand, the resultant Akt phosphorylation was significantly decreased in the presence of excess Tf, suggesting a compromised activation of IR.

### Mitogenic potential assessment of ProINS-Tf and irINS-Tf in MCF-7 Cells

**Figure 4:**
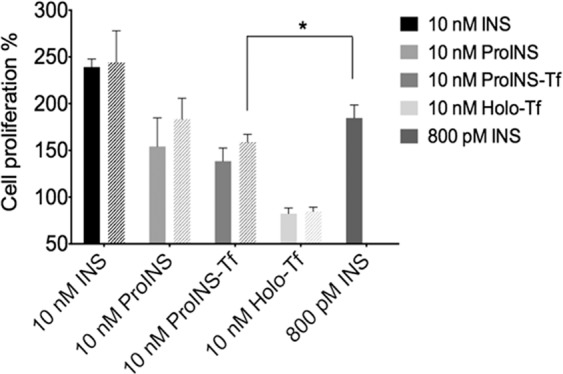
Effects of ProINS-Tf and H4IIE pretreated ProINS-Tf on cell proliferation of MCF-7 cells. MCF-7 cells were treated with 10 nM ProINS-Tf, human INS or holo-Tf for 72 h. Cell viability was determined by MTT assay (n = 3). For each protein, cell proliferation level was also compared between intact protein (filled bar) and H4IIE pretreated protein (bar with strips). *Indicated *p* ≤ 0.05.

In order to address whether the enhanced binding and prolonged retention of irINS-Tf to IR leads to increased mitogenic potential, proliferation assays in MCF-7 cells were performed to compare the mitogenic effect between native INS, ProINS, ProINS-Tf and irINS-Tf. As shown in Fig. 4, compared with the non-treated group, MCF-7 cell proliferation was increased to 239 ± 5% in the 10 nM INS treated group and 138 ± 7% in the 10 nM ProINS-Tf treated group, while no significant increase was observed after 10 nM holo-Tf treatment. In addition, ProINS-Tf elicited similar cell proliferation level as ProINS (154 ± 15%). Since ProINS does not bind IGF-1R^20^, the increased cell proliferation level in both ProINS-Tf and ProINS groups compared to the control group could be due to the intrinsic cell growth stimulatory effects of IR signaling. It was also noticed that H4IIE pretreatment of ProINS-Tf did not induce apparent changes in MCF-7 proliferation. In the treatment groups, the irINS-Tf level from 10 nM ProINS-Tf after 24 h incubation with H4IIE in this set of studies was determined as 800 pM using INS-specific RIA. Clearly, the pretreated ProINS-Tf demonstrated weaker cell proliferation activity than the native INS at the comparable concentration of 800 pM.

### Akt phosphorylation assay in INS resistant cells

**Figure 5:**
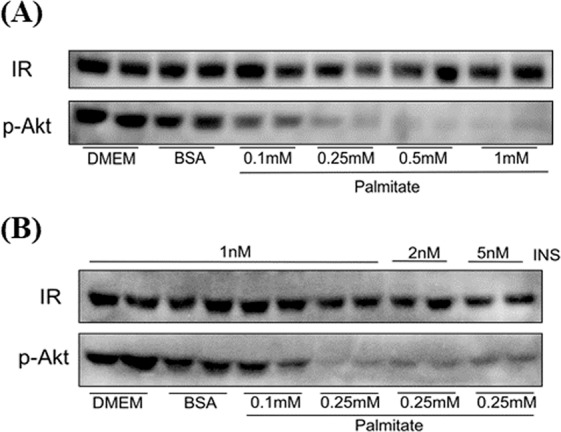
Palmitate induced INS resistance in HepG2 cells. (**A**) Dose-dependent palmitate-induced INS resistance in HepG2 cells. HepG2 cells were incubated with indicated concentration of palmitate complex for 16 h, and then stimulated with 1 nM of INS for 10 min. (**B**) Effect of INS in palmitate treated HepG2 cells. HepG2 cells treated with 0.25 mM of palmitate was stimulated by different concentrations of INS for 10 min.

Treatment of HepG2 cells with palmitate resulted in decreased Akt phosphorylation in response to INS stimulation, without affecting the expression level of IR (Fig. 5A). The induction of INS desensitization by palmitate was dose-dependent, with higher concentration of palmitate resulting in lower p-Akt response to INS. A concentration of 0.25 mM palmitate was chosen for further experiments. At this dose, palmitate-treated cells were INS resistant, but still slightly responsive to INS stimulation in terms of Akt phosphorylation (Fig. 5B).

**Figure 6:**
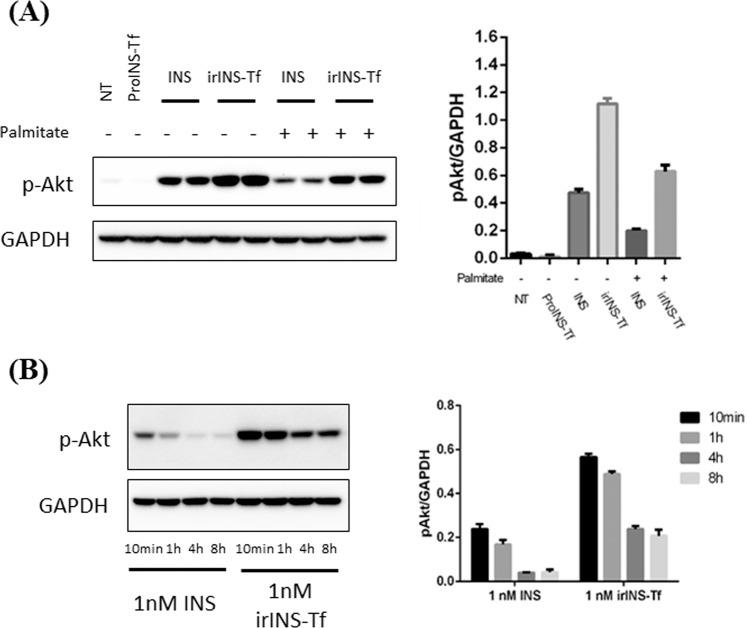
irINS-Tf overcame INS resistance in HepG2 cells. (**A**) Onset Akt phosphorylation in palmitate treated HepG2 cells. HepG2 cells were incubated with or without 0.25 mM of palmitate complex for 16 h, and then stimulated with 1 nM ProINS-Tf, INS, or irINS-Tf for 10 min. The p-Akt intensity detected from different treatment groups was normalized to GAPDH intensity, and the normalized results were plotted in bar graph as shown on the right. (**B**) Time-course Akt phosphorylation in INS resistant HepG2 cells. HepG2 cells with INS resistance were treated with 1 nM of INS or 1 nM irINS-Tf for indicated period of time. Phospho-Akt band density was normalized with corresponding GAPDH band density. Data were presented as the average values with error bars indicating the standard deviation (N = 3). Please see Supplemental Information for the ANOVA comparisons.

Akt phosphorylation stimulated by INS and irINS-Tf was evaluated in palmitate-treated HepG2 cells to compare their abilities on activating IR in an INS resistance model. INS resistance was confirmed in palmitate-treated cells by decreased level of p-Akt induced by INS and irINS-Tf (Fig. 6A). In palmitate-treated cells, p-Akt level induced by 1 nM irINS-Tf was approximately 3-fold higher than the signal induced by 1 nM of native INS. In addition, the Akt phosphorylation level stimulated by irINS-Tf in INS–resistant cells was comparable to the p-Akt level stimulated by INS in normal cells (Fig. 6B). These data indicated that irINS-Tf can overcome palmitate-induced INS resistance in HepG2 cells. (Please see Supplementary Information for detailed statistical analysis).

### Therapeutic efficacy study in NOD mice with INS resistance symptom

**Figure 7:**
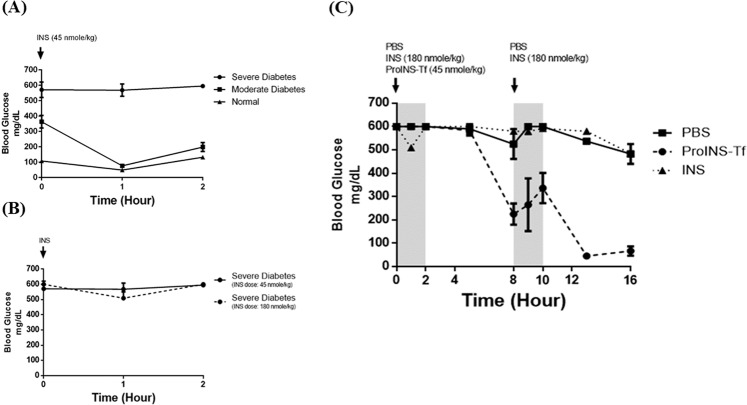
Therapeutic efficacy of ProINS-Tf in NOD mice with severe hyperglycemia. (**A**) Effect of INS in NOD mice with different glycemic conditions. NOD mice were categorized into three groups (severe: blood glucose (BG) above 500 mg/dL, n = 3; moderate: BG between 300–500 mg/dL, n = 3; normal: BG around 100 mg/dL, n = 3) and subcutaneously injected with 45 nmol/kg of INS. BG levels were monitored 1 h and 2 h post injection under free-feeding condition. (**B**) Effect of escalated INS dose in NOD mice with severe hyperglycemia. NOD mice with severe hyperglycemia were subcutaneously injected with either 45 nmol/kg (n = 3) or 180 nmol/kg (n = 3) of INS, and the BG levels were monitored 1 h and 2 h post injection under free-feeding condition. (**C**) Therapeutic effect of a single ProINS-Tf injection in NOD mice with severe hyperglycemia. NOD mice with severe hyperglycemia were subcutaneously injected with a single injection of ProINS-Tf (dose: 45 nmol/kg, n = 4), two injections of INS (each dose: 180 nmole/kg, n = 4) or two injections of PBS, and the blood glucose level was monitored during two 2 h feeding (grey shading)/6 h fasting (no shading) cycles. Readings above 600 mg/dL were presented as 600 mg/dL due to limitation of the detection range. Blood glucose curves reflected average values and the error bars indicated standard deviation. Please see Supplemental Information for the ANOVA comparisons.

Therapeutic efficacy comparison between ProINS-Tf and INS in NOD mice with severe hyperglycemia and insulin resistance (Fig. 7A,B) were performed in a meal challenge study. Significant reduction in blood glucose level was detected starting from 5 h post injection with a dose of 45 nmol/kg of ProINS-Tf administration (Fig. 7C, solid circles), with a 62.5% and 89% reduction at 8 and 16 h post a single injection at the zero time. On the other hand, the blood glucose levels were only reduced by 17% and 5% immediately following each high-dose INS injection (180 nmol/kg) before the start of each feeding period (solid triangle). (Please see Supplementary Information for detailed statistical analysis). These results indicated that, even with much lower dose, ProINS-Tf had a more potent and sustained effect in reducing blood glucose level compared with the native INS, which is consistent with the findings observed in the cell-based pulse-chase and time-course studies (Figs. 1 and 6).

## Discussion

The mechanistic study of ProINS-Tf on binding to IR was carried out on HepG2 cells. HepG2 cells were reported to express extremely low levels of hepatic metabolism enzymes and altered TfR recycling mechanism^15,16^, and showed in our previous study to be ineffective at converting ProINS-Tf to its active form^17^. Using this cell line for studying the active and inactive forms of the fusion protein could avoid the background activity generated by the additional activation of the fusion protein during the course of incubation. HepG2 cells were also shown to express relatively equal amount of IR and TfR^21^, making it a good model to investigate the potential bivalent binding mechanism of irINS-Tf.

When compared to INS for the capability on inducing Akt phosphorylation, active irINS-Tf demonstrated much higher activity under both onset (at 10 min) and prolonged (up to 8 h) conditions (Fig. 1A), suggesting its enhanced ability on activating IR. ProINS-Tf by itself had little activity on Akt phosphorylation and became much more active after the H4IIE cells-mediated conversion^8^. The increased p-Akt levels induced by irINS-Tf could be due to the increased stability of the fusion protein in the dosing medium, and/or the enhanced and sustained binding of irINS-Tf with the IR. To further investigate the action of irINS-Tf, a pulse-chase Akt phosphorylation was performed, in which cells were pulsed with dosing proteins under 4 °C, and then chased in protein-free medium. In this scenario, Akt phosphorylation will be induced by the proteins that remained associated at the cell membrane after the pulse phase, serving as a reliable indication of the prolonged association of the proteins with IR. Compared to INS, whose activity diminished very fast in the pulse-chase experiment, irINS-Tf showed a sustained effect on causing Akt phosphorylation (Fig. 1B), suggesting its prolonged association with IR in addition to the improved stability.

A series of IR competitive binding experiments were conducted to see if the activity on causing Akt phosphorylation was correlated with the binding affinity to IR. ProINS-Tf initially had very low affinity to IR and barely displaced any binding of the radioactive ^125^I-INS tracer at a concentration more than 10-fold higher than the IC_50_ of INS (Fig. 2B). When the fusion protein was pre-incubated with H4IIE cells for activation, its affinity to IR significantly increased compared to the inactive prodrug (Fig. 2B). Since the inactive ProINS-Tf had very minimal binding effect to IR, the binding activity of the partially activated fusion protein mixture can be mostly contributed by the active irINS-Tf. After INS-specific RIA quantification, there was 0.67 nM irINS-Tf generated after the conversion from 10 nM ProINS-Tf, an approximate activation of 6.7%. The IC_50_ of the irINS-Tf could therefore be roughly estimated as 1.24 nM, which was even lower than the estimated IC_50_ of native INS, indicating the active fusion protein possessed higher affinity to IR.

The enhanced affinity of irINS-Tf to IR could be due to the fusion protein acting as a bivalent ligand, simultaneously binding to both the IR and TfR. This possibility is further supported by the effect excess Tf had on the IR binding affinity and INS-mediated activity. As shown in Fig. 3B,C, with the addition of 100-fold Tf to partially block the TfRs on the cell surface, the activity of irINS-Tf on both inducing Akt phosphorylation and binding to IR was significantly reduced, suggesting that the loss of TfR binding compromised the binding of the irINS-Tf to IR. The enhanced binding affinity and prolonged retention to IR observed in irINS-Tf are similar to the binding characteristics reported in INS X10^22^, which raise the concern that irINS-Tf might induce uncontrolled proliferation after long-term use. Therefore, MCF-7 proliferation assays were performed to preliminarily evaluate the mitogenic effect of the both ProINS-Tf and irINS-Tf. MCF-7 is a human breast cancer cell line which expresses insulin-like growth factor 1 receptor (IGF-1R) at about 9-fold higher than that of IR^23^. Hence, in the IGF-1R rich MCF-7 cells, IR activation mediated cell growth is minimal which makes this cell line a suitable *in vitro* model to study the mitogenic potential for INS and INS analogs^24,25^, including ProINS-Tf and irINS-Tf. The results shown in Fig. 4 demonstrate that, after 72 hours of treatment, both ProINS-Tf and irINS-Tf treatments displayed significantly lower cellular proliferation compared with native INS treatment groups. This finding suggests that, compared with native INS, irINS-Tf may have a less binding affinity toward IGF-1R to activate the IGF-1R-mediated cellular proliferation even with an enhanced binding affinity to the IR.

The increased binding affinity of the activated ProINS-Tf to IR makes the fusion protein utilized valuable treatment option for INS-resistant type 2 diabetes, as well as other metabolic diseases. INS resistance sometimes could be compensated by using increased concentration of INS to achieve the original level of response. With the increased activity seen for irINS-Tf, this fusion protein might be able to achieve improved efficacy than the same concentration of INS in INS resistant cells. In palmitate-treated HepG2 cells with INS resistance, although Akt phosphorylation caused by either INS or irINS-Tf was lower in INS-resistant cells than in wild-type cells, irINS-Tf remained more potent than INS in the resistant cells (Fig. 5A). Similar to the Akt phosphorylation assay in normal HepG2 cells, irINS-Tf caused significantly higher levels of p-Akt than INS at both onset and prolonged-treatment timepoints (Fig. 5B). irINS-Tf demonstrated its enhanced binding ability to IR in INS resistant cells by exhibiting a much stronger effect than native INS on the phosphorylation of Akt. The difference between the wild-type and INS-resistant HepG2 cells in Akt phosphorylation induced by irINS-Tf or native INS treatment (Fig. 5A) suggests the greater advantage of irINS-Tf in INS-resistant cells and the potential application of irINS-Tf in restocking IR response to attenuate INS resistance syndromes.

The comparison of the therapeutic efficacy between INS and ProINS-Tf fusion protein was also evaluated in an animal model with INS resistance symptoms. It has been reported that INS resistance could be developed in nonobese diabetic (NOD) mouse and the condition varies with the severity of its hyperglycemia^26,27^. We confirmed the development of INS resistance in NOD mice with severe hyperglycemia by observing minimal response to high doses of INS treatment (Fig. 7A,B). In mice with severe diabetes, two INS injections at high dose failed to lower the BG concentration to the normal range, suggesting the existence of a considerable level of INS resistance (Fig. 7C, solid triangles). On the other hand, ProINS-Tf at the low dose was able to manage the BG level during an extended period of time with one single injection at the initial point (Fig. 7C, solid circles). The lag-time for activation, liver-preferential activity, and long-lasting BG-lowering effect of ProINS-Tf in INS-resistant NOD mice were similar to that observed in non-INS-resistant diabetes mouse models as described in our previous publications^7,8,28^. This finding indicates that a normal glycemic regulation efficacy of ProINS-Tf can be achieved in INS-resistant diabetic NOD mice. Therefore, both *in vitro* and *in vivo* results suggest that irINS-Tf could achieve the same or better glycemic control with less dose than native INS in treating INS-resistant diabetes.

In summary, the binding mechanism of liver-activated ProINS-Tf, i.e., irINS-Tf, was elucidated in this report. As compared to INS, the active irINS-Tf possessed higher affinity to IR through its bivalent binding to both IR and TfR. With assistance of TfR binding, irINS-Tf was able to achieve improved binding affinity and prolonged activation of IR. The active fusion protein also showed high potency in INS-resistant cells and exhibited a prolonged glucose-lowering effect in INS resistant NOD mice. Taken together, these results demonstrate the potential application of ProINS-Tf as an INS analog for the prevention or treatment of type 2 diabetes and other INS resistance-associated diseases.

## Supplementary information

Supplemental Information.
