# Supplementary material for: Enhanced insulin receptor interaction by a bifunctional insulin-transferrin fusion protein: an approach to overcome insulin resistance
Source: Sci Rep. 2020 May 7;10:7724. doi: 10.1038/s41598-020-64731-9 (PMC7206000; doi:10.1038/s41598-020-64731-9)
Supplement: Supplementary file 1 — Supplemental Information. [file 41598_2020_64731_MOESM1_ESM.pdf]

Supplementary Information

Enhanced insulin receptor interaction by a bifunctional insulin-transferrin fusion protein: an approach to overcome insulin resistance.

Yuqian Liu[1†], Hsuan-Yao Wang[1†], Juntang Shao[1,2], Jennica L. Zaro[1], and Wei-Chiang Shen[1*]

[1]Department of Pharmacology and Pharmaceutical Sciences, University of Southern California School of Pharmacy, 1985 Zonal Ave, Los Angeles, CA 90089-9121

[2]School of Basic Medical Sciences, Anhui Medical University, Hefei 230032, China
†These authors contributed equally to this work.
*Email: weishen@usc.edu

Summary

The Supplementary Information contains *(a)* the detailed statistical analysis for data presented in Figures 6 and 7 of the manuscript (Supplementary Tables 1-5), and *(b)* the Supplementary Figure Legends and original blots for the results shown in Figures 1, 3, 5, and 6 of the manuscript (Supplementary Figures 1-4).

(a) Detailed Statistical Analysis for Figures 6 and 7 of the Manuscript

Supplementary Table 1: One-way ANOVA Tukey's multiple comparisons test result for data presented in Figure 6A

Number of families	1		** represent p<0.01						
Number of comparisons per family	6		**** represent p<0.0001						
Alpha	0.05								
Tukey's multiple comparisons test	Mean Diff.	95% CI of diff.	Significant?	Summary					
1 nM INS vs. 1 nM irINS-Tf	-0.6438	-0.7312 to -0.5564	Yes	****					
1 nM INS vs. Palmitate-treated 1nM INS	0.2733	0.1859 to 0.3607	Yes	****					
1 nM INS vs. Palmitate-treated 1nM irINS-Tf	-0.1563	-0.2437 to -0.06888	Yes	**					
1 nM irINS-Tf vs. Palmitate-treated 1nM INS	0.9171	0.8297 to 1.005	Yes	****					
1 nM irINS-Tf vs. Palmitate-treated 1nM irINS-Tf	0.4875	0.4001 to 0.5749	Yes	****					
Palmitate-treated 1nM INS vs. Palmitate-treated 1nM irINS-Tf	-0.4296	-0.5170 to -0.3422	Yes	****					
Test details	Mean 1	Mean 2	Mean Diff.	SE of diff.	n1	n2	q	DF	
1 nM INS vs. 1 nM irINS-Tf	0.4733	1.117	-0.6438	0.0273	3	3	33.36	8	
1 nM INS vs. Palmitate-treated 1nM INS	0.4733	0.2	0.2733	0.0273	3	3	14.16	8	
1 nM INS vs. Palmitate-treated 1nM irINS-Tf	0.4733	0.6296	-0.1563	0.0273	3	3	8.098	8	
1 nM irINS-Tf vs. Palmitate-treated 1nM INS	1.117	0.2	0.9171	0.0273	3	3	47.52	8	
1 nM irINS-Tf vs. Palmitate-treated 1nM irINS-Tf	1.117	0.6296	0.4875	0.0273	3	3	25.26	8	
Palmitate-treated 1nM INS vs. Palmitate-treated 1nM irINS-Tf	0.2	0.6296	-0.4296	0.0273	3	3	22.26	8	

Supplementary Table 2: Raw data presented in Figure 6B for the ANOVA analysis

Test details	Mean 1	Mean 2	Mean Diff.	SE of diff.	N1	N2	q	DF
1 nM INS:10 min vs. 1 nM INS:1 h	0.2392	0.1686	0.07057	0.01443	3	3	6.914	16
1 nM INS:10 min vs. 1 nM INS:4 h	0.2392	0.04017	0.199	0.01443	3	3	19.5	16
1 nM INS:10 min vs. 1 nM INS:8 hr	0.2392	0.04144	0.1977	0.01443	3	3	19.37	16
1 nM INS:10 min vs. 1 nM irINS-Tf:10 min	0.2392	0.5657	-0.3265	0.01443	3	3	31.99	16
1 nM INS:10 min vs. 1 nM irINS-Tf:1 h	0.2392	0.4882	-0.249	0.01443	3	3	24.4	16
1 nM INS:10 min vs. 1 nM irINS-Tf:4 h	0.2392	0.238	0.001156	0.01443	3	3	0.1133	16
1 nM INS:10 min vs. 1 nM irINS-Tf:8 hr	0.2392	0.208	0.03119	0.01443	3	3	3.056	16
1 nM INS:1 h vs. 1 nM INS:4 h	0.1686	0.04017	0.1284	0.01443	3	3	12.58	16
1 nM INS:1 h vs. 1 nM INS:8 hr	0.1686	0.04144	0.1272	0.01443	3	3	12.46	16
1 nM INS:1 h vs. 1 nM irINS-Tf:10 min	0.1686	0.5657	-0.3971	0.01443	3	3	38.9	16
1 nM INS:1 h vs. 1 nM irINS-Tf:1 h	0.1686	0.4882	-0.3196	0.01443	3	3	31.31	16
1 nM INS:1 h vs. 1 nM irINS-Tf:4 h	0.1686	0.238	-0.06941	0.01443	3	3	6.8	16
1 nM INS:1 h vs. 1 nM irINS-Tf:8 hr	0.1686	0.208	-0.03937	0.01443	3	3	3.857	16
1 nM INS:4 h vs. 1 nM INS:8 hr	0.04017	0.04144	-0.001277	0.01443	3	3	0.1251	16
1 nM INS:4 h vs. 1 nM irINS-Tf:10 min	0.04017	0.5657	-0.5255	0.01443	3	3	51.49	16
1 nM INS:4 h vs. 1 nM irINS-Tf:1 h	0.04017	0.4882	-0.448	0.01443	3	3	43.9	16
1 nM INS:4 h vs. 1 nM irINS-Tf:4 h	0.04017	0.238	-0.1978	0.01443	3	3	19.38	16
1 nM INS:4 h vs. 1 nM irINS-Tf:8 hr	0.04017	0.208	-0.1678	0.01443	3	3	16.44	16
1 nM INS:8 hr vs. 1 nM irINS-Tf:10 min	0.04144	0.5657	-0.5242	0.01443	3	3	51.36	16
1 nM INS:8 hr vs. 1 nM irINS-Tf:1 h	0.04144	0.4882	-0.4468	0.01443	3	3	43.77	16
1 nM INS:8 hr vs. 1 nM irINS-Tf:4 h	0.04144	0.238	-0.1966	0.01443	3	3	19.26	16
1 nM INS:8 hr vs. 1 nM irINS-Tf:8 hr	0.04144	0.208	-0.1665	0.01443	3	3	16.32	16
1 nM irINS-Tf:10 min vs. 1 nM irINS-Tf:1 h	0.5657	0.4882	0.07748	0.01443	3	3	7.591	16
1 nM irINS-Tf:10 min vs. 1 nM irINS-Tf:4 h	0.5657	0.238	0.3277	0.01443	3	3	32.1	16
1 nM irINS-Tf:10 min vs. 1 nM irINS-Tf:8 hr	0.5657	0.208	0.3577	0.01443	3	3	35.05	16
1 nM irINS-Tf:1 h vs. 1 nM irINS-Tf:4 h	0.4882	0.238	0.2502	0.01443	3	3	24.51	16
1 nM irINS-Tf:1 h vs. 1 nM irINS-Tf:8 hr	0.4882	0.208	0.2802	0.01443	3	3	27.46	16
1 nM irINS-Tf:4 h vs. 1 nM irINS-Tf:8 hr	0.238	0.208	0.03004	0.01443	3	3	2.943	16

Supplementary Table 3: Two-way ANOVA Tukey’s multiple comparisons test result for data presented in Figure 6B.

Number of families	1			** represent p<0.01
Number of comparisons per family	28			**** represent p<0.0001
Alpha	0.05			ns represent non-significant
Tukey's multiple comparisons test	Mean Diff.	95% CI of diff.	Significant?	Summary
1 nM INS:10 min vs. 1 nM INS:1 h	0.07057	0.02059 to 0.1205	Yes	**
1 nM INS:10 min vs. 1 nM INS:4 h	0.199	0.1490 to 0.2490	Yes	****
1 nM INS:10 min vs. 1 nM INS:8 hr	0.1977	0.1477 to 0.2477	Yes	****
1 nM INS:10 min vs. 1 nM irINS-Tf:10 min	-0.3265	-0.3765 to -0.2765	Yes	****
1 nM INS:10 min vs. 1 nM irINS-Tf:1 h	-0.249	-0.2990 to -0.1991	Yes	****
1 nM INS:10 min vs. 1 nM irINS-Tf:4 h	0.001156	-0.04882 to 0.05113	No	ns
1 nM INS:10 min vs. 1 nM irINS-Tf:8 hr	0.03119	-0.01878 to 0.08117	No	ns
1 nM INS:1 h vs. 1 nM INS:4 h	0.1284	0.07846 to 0.1784	Yes	****
1 nM INS:1 h vs. 1 nM INS:8 hr	0.1272	0.07718 to 0.1771	Yes	****
1 nM INS:1 h vs. 1 nM irINS-Tf:10 min	-0.3971	-0.4471 to -0.3471	Yes	****
1 nM INS:1 h vs. 1 nM irINS-Tf:1 h	-0.3196	-0.3696 to -0.2696	Yes	****
1 nM INS:1 h vs. 1 nM irINS-Tf:4 h	-0.06941	-0.1194 to -0.01944	Yes	**
1 nM INS:1 h vs. 1 nM irINS-Tf:8 hr	-0.03937	-0.08934 to 0.01060	No	ns
1 nM INS:4 h vs. 1 nM INS:8 hr	-0.001277	-0.05125 to 0.04870	No	ns
1 nM INS:4 h vs. 1 nM irINS-Tf:10 min	-0.5255	-0.5755 to -0.4755	Yes	****
1 nM INS:4 h vs. 1 nM irINS-Tf:1 h	-0.448	-0.4980 to -0.3981	Yes	****
1 nM INS:4 h vs. 1 nM irINS-Tf:4 h	-0.1978	-0.2478 to -0.1479	Yes	****
1 nM INS:4 h vs. 1 nM irINS-Tf:8 hr	-0.1678	-0.2178 to -0.1178	Yes	****
1 nM INS:8 hr vs. 1 nM irINS-Tf:10 min	-0.5242	-0.5742 to -0.4743	Yes	****
1 nM INS:8 hr vs. 1 nM irINS-Tf:1 h	-0.4468	-0.4967 to -0.3968	Yes	****
1 nM INS:8 hr vs. 1 nM irINS-Tf:4 h	-0.1966	-0.2465 to -0.1466	Yes	****
1 nM INS:8 hr vs. 1 nM irINS-Tf:8 hr	-0.1665	-0.2165 to -0.1166	Yes	****
1 nM irINS-Tf:10 min vs. 1 nM irINS-Tf:1 h	0.07748	0.02751 to 0.1275	Yes	**
1 nM irINS-Tf:10 min vs. 1 nM irINS-Tf:4 h	0.3277	0.2777 to 0.3776	Yes	****
1 nM irINS-Tf:10 min vs. 1 nM irINS-Tf:8 hr	0.3577	0.3077 to 0.4077	Yes	****
1 nM irINS-Tf:1 h vs. 1 nM irINS-Tf:4 h	0.2502	0.2002 to 0.3002	Yes	****
1 nM irINS-Tf:1 h vs. 1 nM irINS-Tf:8 hr	0.2802	0.2303 to 0.3302	Yes	****
1 nM irINS-Tf:4 h vs. 1 nM irINS-Tf:8 hr	0.03004	-0.01993 to 0.08001	No	ns

Supplementary Table 4: Raw data presented in Figure 7C for the ANOVA analysis

Test details	Mean1	Mean 2	Mean Diff.	SE of diff.	N1	N2	q	DF
0 h								
PBS vs. ProINS-Tf	600	600	0	25.54	4	4	0	81
PBS vs. INS	600	600	0	25.54	4	4	0	81
ProINS-Tf vs. INS	600	600	0	25.54	4	4	0	81
1 h								
PBS vs. ProINS-Tf	600	600	0	25.54	4	4	0	81
PBS vs. INS	600	508.8	91.25	25.54	4	4	5.052	81
ProINS-Tf vs. INS	600	508.8	91.25	25.54	4	4	5.052	81
2 h								
PBS vs. ProINS-Tf	600	600	0	25.54	4	4	0	81
PBS vs. INS	600	600	0	25.54	4	4	0	81
ProINS-Tf vs. INS	600	600	0	25.54	4	4	0	81
5 h								
PBS vs. ProINS-Tf	590.3	583	7.25	25.54	4	4	0.4014	81
PBS vs. INS	590.3	600	-9.75	25.54	4	4	0.5398	81
ProINS-Tf vs. INS	583	600	-17	25.54	4	4	0.9412	81
8 h								
PBS vs. ProINS-Tf	525	225	300	25.54	4	4	16.61	81
PBS vs. INS	525	580.3	-55.25	25.54	4	4	3.059	81
ProINS-Tf vs. INS	225	580.3	-355.3	25.54	4	4	19.67	81
9 h								
PBS vs. ProINS-Tf	600	264.8	335.3	25.54	4	4	18.56	81
PBS vs. INS	600	579.3	20.75	25.54	4	4	1.149	81
ProINS-Tf vs. INS	264.8	579.3	-314.5	25.54	4	4	17.41	81
10 h								
PBS vs. ProINS-Tf	600	336.3	263.8	25.54	4	4	14.6	81
PBS vs. INS	600	592	8	25.54	4	4	0.4429	81
ProINS-Tf vs. INS	336.3	592	-255.8	25.54	4	4	14.16	81
13 h								
PBS vs. ProINS-Tf	537.3	45.25	492	25.54	4	4	27.24	81
PBS vs. INS	537.3	579.5	-42.25	25.54	4	4	2.339	81
ProINS-Tf vs. INS	45.25	579.5	-534.3	25.54	4	4	29.58	81
16 h								
PBS vs. ProINS-Tf	482.5	66.75	415.8	25.54	4	4	23.02	81
PBS vs. INS	482.5	484.5	-2	25.54	4	4	0.1107	81
ProINS-Tf vs. INS	66.75	484.5	-417.8	25.54	4	4	23.13	81

Supplementary Table 5: Two-way ANOVA Tukey's multiple comparisons test result for data presented in Figure 7C.

Number of families	9		** represent p<0.01	
Number of comparisons per family	3		**** represent p<0.0001	
Alpha	0.05		ns represent non-significant	
Tukey's multiple comparisons test	Mean Diff.	95% CI of diff.	Significant?	Summary
0 h				
PBS vs. ProINS-Tf	0	-60.99 to 60.99	No	ns
PBS vs. INS	0	-60.99 to 60.99	No	ns
ProINS-Tf vs. INS	0	-60.99 to 60.99	No	ns
1 h				
PBS vs. ProINS-Tf	0	-60.99 to 60.99	No	ns
PBS vs. INS	91.25	30.26 to 152.2	Yes	**
ProINS-Tf vs. INS	91.25	30.26 to 152.2	Yes	**
2 h				
PBS vs. ProINS-Tf	0	-60.99 to 60.99	No	ns
PBS vs. INS	0	-60.99 to 60.99	No	ns
ProINS-Tf vs. INS	0	-60.99 to 60.99	No	ns
5 h				
PBS vs. ProINS-Tf	7.25	-53.74 to 68.24	No	ns
PBS vs. INS	-9.75	-70.74 to 51.24	No	ns
ProINS-Tf vs. INS	-17	-77.99 to 43.99	No	ns
8 h				
PBS vs. ProINS-Tf	300	239.0 to 361.0	Yes	****
PBS vs. INS	-55.25	-116.2 to 5.736	No	ns
ProINS-Tf vs. INS	-355.3	-416.2 to -294.3	Yes	****
9 h				
PBS vs. ProINS-Tf	335.3	274.3 to 396.2	Yes	****
PBS vs. INS	20.75	-40.24 to 81.74	No	ns
ProINS-Tf vs. INS	-314.5	-375.5 to -253.5	Yes	****
10 h				
PBS vs. ProINS-Tf	263.8	202.8 to 324.7	Yes	****
PBS vs. INS	8	-52.99 to 68.99	No	ns
ProINS-Tf vs. INS	-255.8	-316.7 to -194.8	Yes	****
13 h				
PBS vs. ProINS-Tf	492	431.0 to 553.0	Yes	****
PBS vs. INS	-42.25	-103.2 to 18.74	No	ns
ProINS-Tf vs. INS	-534.3	-595.2 to -473.3	Yes	****
16 h				
PBS vs. ProINS-Tf	415.8	354.8 to 476.7	Yes	****
PBS vs. INS	-2	-62.99 to 58.99	No	ns
ProINS-Tf vs. INS	-417.8	-478.7 to -356.8	Yes	****

(b) **Supplementary Figure Legends and Original Blots for Figures 1, 3, 5, and 6 of the Manuscript**

Supplementary Figure Legends

Supplementary Figure 1. Active irINS-Tf exhibited enhanced and prolonged effect on inducing Akt phosphorylation in HepG2 cells (Original blot of Figure 1 in the manuscript). (A, B) Time-course Akt phosphorylation in HepG2 cells. Starved HepG2 cells were treated for indicated period of time with 1 nM of INS, 10 nM of INS, or irINS-Tf converted from10 nM of ProINS-Tf. (C) Pulse-chase Akt phosphorylation assay in HepG2 cells. Starved HepG2 cells were treated with ice-cold dosing solution containing 10 nM of INS or irINS-Tf converted from 10 nM of ProINS-Tf for 30 min at 4°C, and then chased in DMEM only medium for indicated period of time at 37°C. After treatment, cells lysates were subjected to Western blot analysis utilizing anti-pAkt antibody or anti-GAPDH antibody as indicated. Solid line boxes indicate the edges of the blot; Dashed line boxes indicate the cropped areas presented in **Figure 1(A) and (B)** in the manuscript.

Supplementary Figure 2. Effect of Tf competition on the IR binding of INS and irINS-Tf in HepG2 cells (Original blot of Figure 3(C) in the manuscript). Akt phosphorylation assay with Tf competition in HepG2 cells. Starved HepG2 cells were treated for 10 min with 10 nM of INS or irINS-Tf converted from 10 nM of ProINS-Tf, with or without addition of 100-fold Tf (1μM). After treatment, cells lysates were subjected to Western blot analysis utilizing anti-pAkt antibody or anti-GAPDH antibody as indicated. Solid line boxes indicate the edges of the blot; Dashed line boxes indicate the cropped areas presented in **Figure 3(C)** in the manuscript.

Supplementary Figure 3. Palmitate induced INS resistance in HepG2 cells (Original blot of Figure 5 in the manuscript). (A) Dose-dependent palmitate-induced INS resistance in HepG2 cells. HepG2 cells were incubated with indicated concentration of palmitate complex for 16 h, and then stimulated with 1 nM of INS for 10 min. (B) Effect of INS in palmitate treated HepG2 cells. HepG2 cells treated with 0.25 mM of palmitate was stimulated by different concentrations of INS for 10 min. After treatment, cells lysates were subjected to Western blot analysis utilizing anti-IR antibody or anti-pAkt antibody as indicated. Solid line boxes indicate the edges of the blot; Dashed line boxes indicate the cropped areas presented in **Figure 5(A) and 5(B)** in the manuscript, respectively.

Supplementary Figure 4. irINS-Tf overcame INS resistance in HepG2 cells (Original blot of Figure 6 in the manuscript). (A,B) Onset Akt phosphorylation in palmitate treated HepG2 cells. HepG2 cells were incubated with or without 0.25 mM of palmitate complex for 16 h, and then stimulated with 1nM ProINS-Tf, INS, or irINS-Tf for 10 min. (C,D) Time-course Akt phosphorylation in INS resistant HepG2 cells. HepG2 cells with INS resistance were treated with 1 nM of INS or 1 nM irINS-Tf for indicated period of time. After treatment, cells lysates were subjected to Western blot analysis utilizing anti-pAkt antibody (A,C) or anti-GAPDH antibody (B,D) as indicated. Solid line boxes indicate the edges of the blot; Dashed line boxes indicate the cropped areas presented in **Figure 6(A) and 6(B)** in the manuscript, respectively.

Suppl. Fig.1(A)
(Fig. 1(A), top panel,
in manuscript)

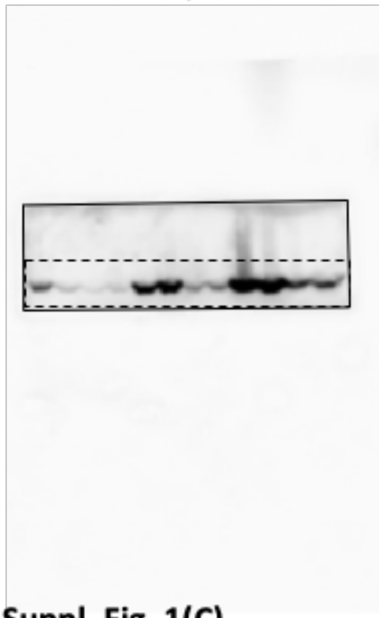

Suppl. Fig. 1(B)
(Fig. 1(A), bottom panel,
in manuscript)

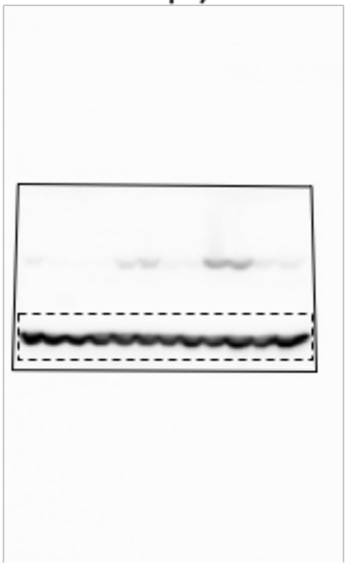

Suppl. Fig. 1(C)
(Fig. 1(B) in manuscript)

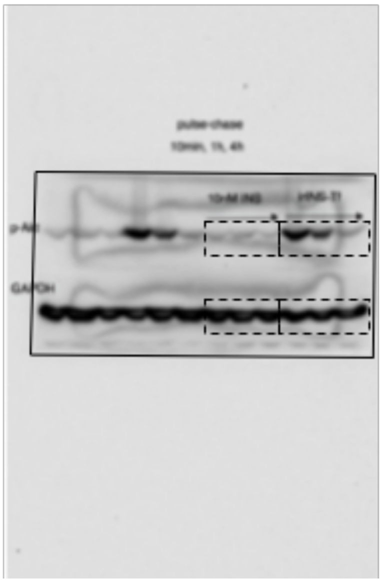

Supplementary Figure 1. Active irINS-Tf exhibited enhanced and prolonged effect on inducing Akt phosphorylation in HepG2 cells (Original blot of Figure 1 in the manuscript).

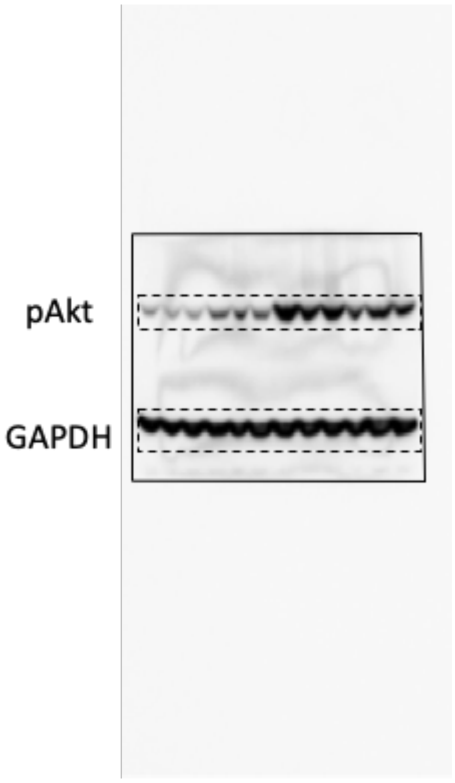

Supplementary Figure 2. Effect of Tf competition on the IR binding of INS and irINS-Tf in HepG2 cells (Original blot of Figure 3(C) in the manuscript).

Supp Fig. 3(A)
(Fig. 5(A) in manuscript)

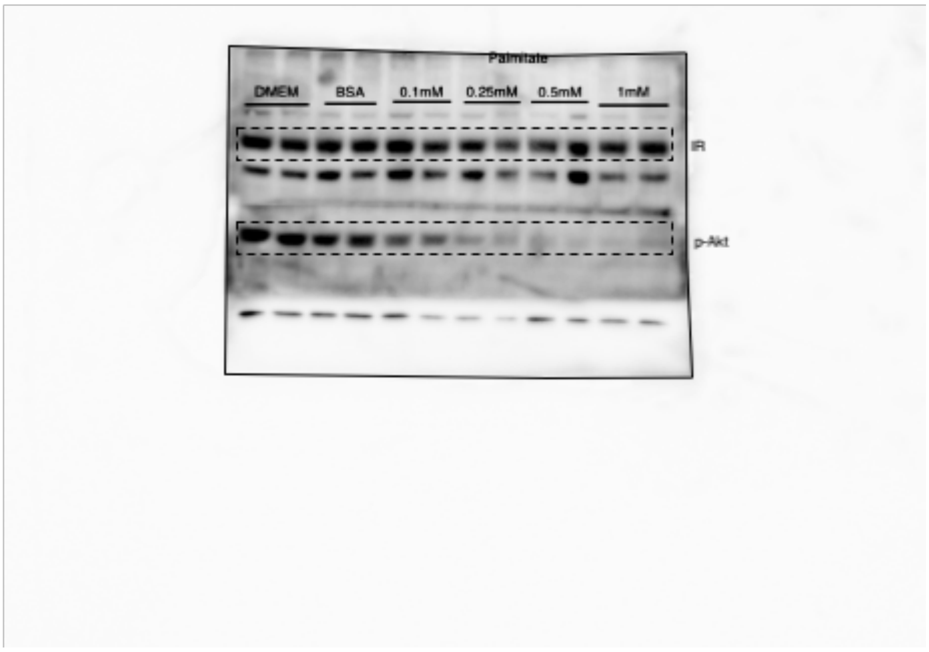

Supp Fig. 3(B)
(Fig. 5(B) in manuscript)

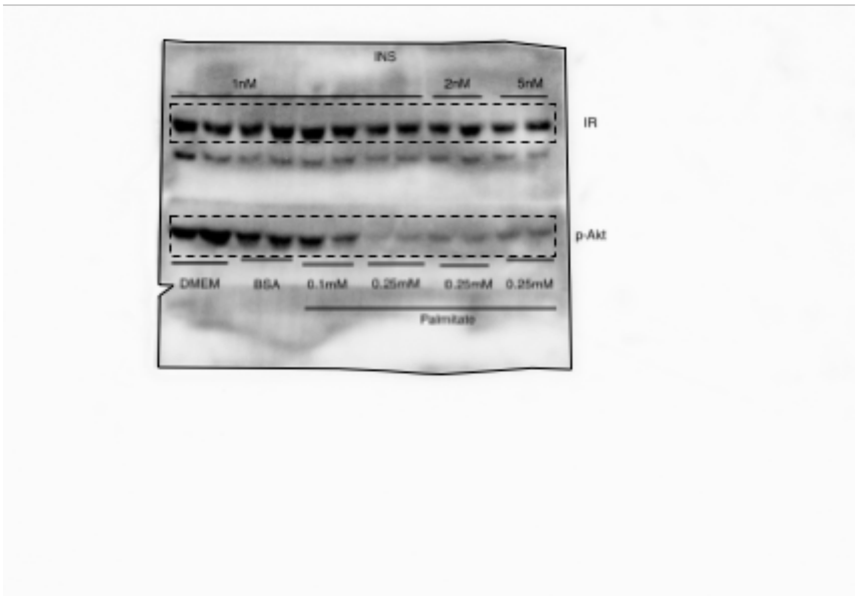

Supplementary Figure 3. Palmitate induced INS resistance in HepG2 cells (Original blot of Figure 5 in the manuscript).

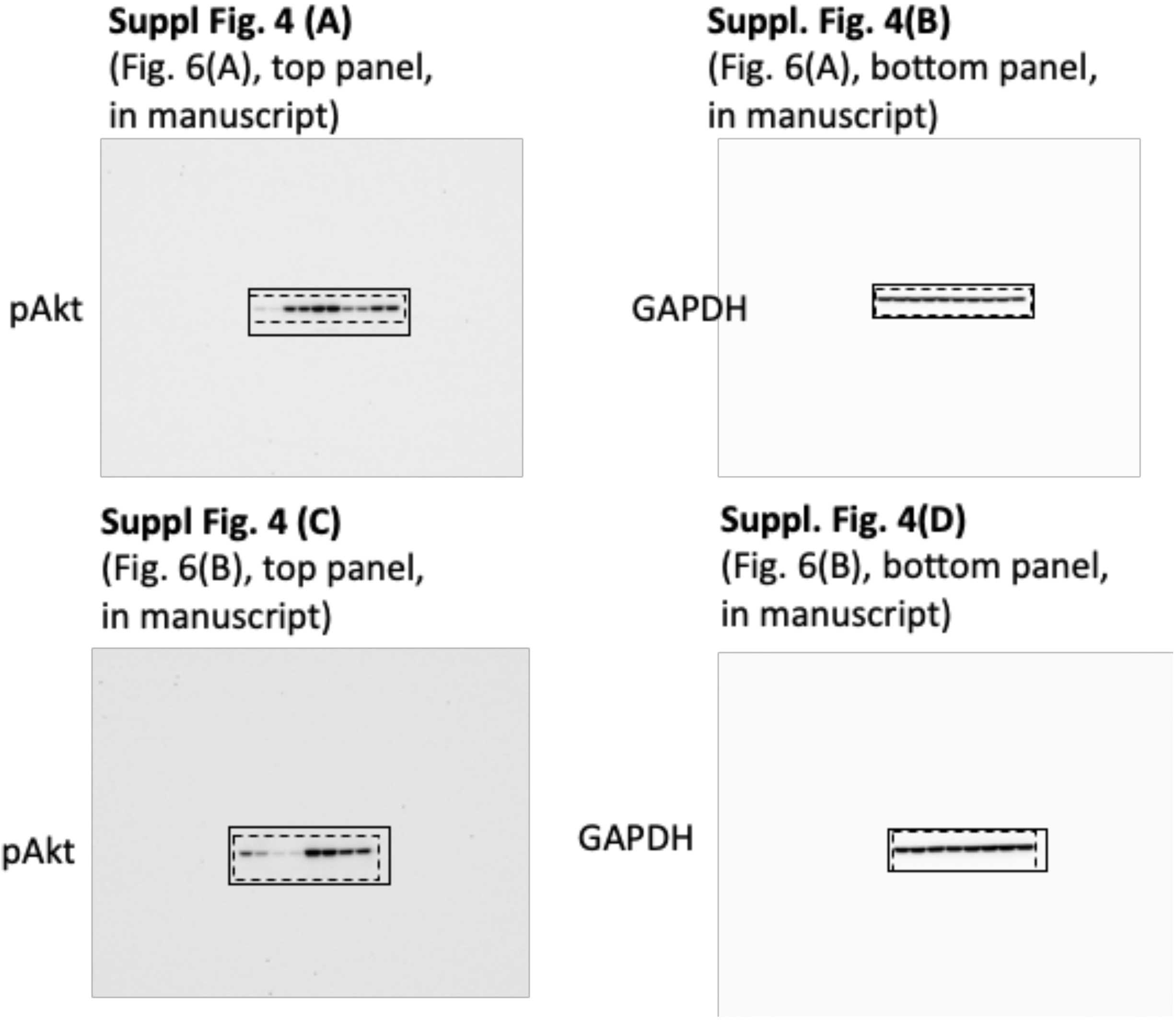

Supplementary Figure 4. irINS-Tf overcame INS resistance in HepG2 cells (Original blot of Figure 6 in the manuscript).
